# Behind the Mask: How the World Survived SARS, the First Epidemic of the 21st Century

**DOI:** 10.3201/eid1112.051058

**Published:** 2005-12

**Authors:** Mehran S. Massoudi

**Affiliations:** * Centers for Disease Control and Prevention, Atlanta, Georgia, USA

**Keywords:** SARS, book review, epidemic

Behind the Mask: How the World Survived SARS, the First Epidemic of the Twenty-First Century recounts the outbreak of Severe Acute Respiratory Syndrome (SARS) that swept through much of the world, especially Asia, in 2003. The author does a superb job of telling the reader about what was occurring before SARS appeared, what happened during the outbreak, and what efforts are underway to prevent its return. The author has blended research results and interviews with frontline staff, particularly healthcare providers, into 20 nicely interlaced chapters. The stage for this commentary is a timeline of events, starting November 16, 2002, with the first known case of SARS in Guangdong Province, China, and ending in December 2003–January 2004 with 4 cases of SARS and the slaughtering of ≈10,000 civets in Guangdong Province. Where possible, the author avoided the use of medical terms and jargon and provides helpful lay translations where their use was unavoidable. As a result, the book is accessible to readers both inside and outside the healthcare arena.

The information presented in the book is, for the most part, current and accurate; different views and beliefs are presented when necessary. There are minor typographical mistakes as well as a few incorrect statements, such as in chapter 1, page 6, where the author refers to past public health efforts to eradicate viruses. The author states that smallpox virus and poliovirus have both been eradicated and that both are now bioterrorism agents. However, despite tremendous progress through efforts of many governments and public and private entities, poliovirus has yet to be eradicated and is not regarded as a bioterrorism agent at this time.

With regard to SARS, however, the author successfully portrays the human side of the outbreak response—a response heralded as unparalleled by many of the involved officials. Dr Carlo Urbani, the World Health Organization (WHO) physician in Vietnam who worked tirelessly and who was an eventual casualty of SARS, is among the many heroes who are featured in this book. Lesser-known facts, such as the thought processes that led to identifying SARS, are also provided. For example, before the SARS coronavirus was shown to be the causative agent, the outbreak was thought to be caused by either avian influenza or chlamydia. The reader is made aware of all of the challenges posed by the SARS virus, such as the delay in recognizing that there was an outbreak, the difficulties in diagnosing and reporting the disease and obtaining specimens, the breadth and scope of national and international collaboration and coordination, and not knowing the causative agent.

This book does a nice job of giving readers a flavor of the experiences faced by persons at WHO, persons at the country ministry level, individual healthcare providers, and SARS patients. I highly recommend this book, especially to anyone who was not directly involved in the SARS outbreak response; they too can share the experience of the global community response to a disease that was first recognized in 1 province of China.

